# Association Between Medicaid Waivers and Medicaid Disenrollment Among Autistic Adolescents During the Transition to Adulthood

**DOI:** 10.1001/jamanetworkopen.2023.2768

**Published:** 2023-03-13

**Authors:** Meghan E. Carey, Sha Tao, Kaitlin H. Koffer Miller, Steven C. Marcus, David S. Mandell, Andrew J. Epstein, Lindsay L. Shea

**Affiliations:** 1 A. J. Drexel Autism Institute, Drexel University, Philadelphia, Pennsylvania; 2 School of Special Policy and Practice, University of Pennsylvania, Philadelphia; 3 Center for Mental Health, Perelman School of Medicine, University of Pennsylvania, Philadelphia; 4 Data Analytics, Medicus Economics, Milton, Massachusetts

## Abstract

This cohort study examines whether Medicaid waivers were associated with a reduced risk of Medicaid disenrollment among autistic adolescents who are transitioning to adulthood.

## Introduction

Autistic youths are more likely than their nonautistic peers to disenroll from Medicaid as they enter adulthood due to eligibility changes and lack of appropriate Medicaid services.^1^ States can provide services through waivers that target eligibility criteria based on age and/or diagnosis for specific populations.^2^ We examined whether waivers were associated with reduced risk of Medicaid disenrollment among autistic adolescents entering adulthood.

## Methods

This cohort study was approved by the Drexel University Institutional Review Board, which waived the need for informed consent owing to the secondary data analysis. The study followed the STROBE reporting guideline.

We extracted data from Medicaid for the period 2008 to 2016 from 47 states and Washington, DC (Arizona, Rhode Island, and Vermont utilize section 1115 of the Social Security Act vs traditional waivers and were therefore excluded). Individuals were included if they: (1) had 12 or more consecutive months of Medicaid enrollment, (2) had at least 1 inpatient or at least 2 other claims with an autism spectrum disorder (ASD) diagnosis code,^3^ and (3) were aged 14 to 26 years during the study period.

We constructed longitudinal analytic data sets at the person-month level, combining individual-month–level information on Medicaid eligibility and service use with state-level information on Medicaid programs and procedures. The binary outcome was first disenrollment while enrolled in Medicaid.^1^ The exposure was a time-varying categorical measure of waiver availability constructed as residence in a state with an active ASD-specific 1915(c) waiver (hereinafter ASD-specific), a non–ASD-specific 1915(c) waiver (hereinafter other), or no waiver. The exposure was interacted with age in a given month to assess variation in the waiver availability–disenrollment association by age. Beneficiary characteristics served as covariates, along with state of residence and calendar year. Characteristics were compared between continuously enrolled and disenrolled individuals using χ^2^ tests with a 2-sided α of .05. Risk of disenrollment was assessed using marginal predicted probabilities as generated from an adjusted person-month discrete-time proportional hazards model. Additional details are provided in the eMethods in Supplement 1.

## Results

This study included 133 955 autistic individuals: 14 739 who had disenrolled in Medicaid and 119 216 who were continuously enrolled; the mean (SD) age for the total cohort was 16.5 (3.4) years. A higher proportion of disenrolled vs continuously enrolled individuals were Medicaid-eligible due to poverty (22.5% vs 5.3%, respectively); a lower proportion were Medicaid-eligible because of disability (47.4% vs 75.6%, respectively) (Table). A smaller proportion of disenrolled vs continuously enrolled individuals were dually enrolled in Medicare (5.8% vs 14.8%, respectively), and similar proportions of individuals had ASD-specific waivers available in their state (5.6% vs 4.1%, respectively).

**Table.** Characteristics of Medicaid Beneficiaries on the Autism Spectrum by Medicaid Enrollment Status, 2008 to 2016^a^

Characteristic	Medicaid enrollment status		*P* value
	Disenrolled (n = 14 739)	Continuous enrollment (n = 119 216)	
Age, y			
14-17	11 507 (78.1)	80 646 (67.6)	<.001
18-21	2621 (17.8)	24 018 (20.2)	
22-26	611 (4.1)	14 552 (12.2)	
Sex			
Male	10 998 (74.6)	93 471 (78.4)	<.001
Female	3741 (25.4)	25 745 (21.6)	
Race and ethnicity			
American Indian or Alaska Native	134 (0.9)	894 (0.8)	<.001
Asian or Hawaiian or other Pacific Islander	280 (1.9)	3917 (3.3)	
Black	3172 (21.5)	17 903 (15.0)	
Hispanic or Latino	1188 (8.1)	14 439 (12.1)	
White	9360 (63.5)	65 432 (54.9)	
Multiracial	104 (0.7)	1116 (0.9)	
Missing	501 (3.4)	15 515 (13.0)	
Eligibility group^b^			
Poverty	4839 (32.8)	6331 (5.3)	<.001
Disability	6366 (43.2)	89 750 (75.3)	
Other	3181 (21.6)	18 094 (15.2)	
Missing	353 (2.4)	5041 (4.2)	
Coverage type^b^			
FFS or PCCM only	4911 (33.3)	30 509 (25.6)	<.001
Any CMC	9828 (66.7)	88 629 (74.3)	
Missing	0	78 (0.1)	
Medicare dual enrollment	768 (5.2)	17 020 (14.3)	<.001
Available waiver			
ASD-specific waiver^c^	1380 (9.4)	4976 (4.2)	<.001
Other 1915(c) waiver^d^	12 876 (87.4)	110 056 (92.3)	
No waiver	483 (3.3)	4184 (3.5)	

Abbreviations: ASD, autism spectrum disorder; CMC, comprehensive managed care; FFS, fee-for-service; PCCM, primary care case management.

^a^ Data are presented as No. (%) of Medicaid beneficiaries. Percentages are rounded and therefore may not sum to 100.

^b^ Variables were constructed using status in the immediate prior month.

^c^ Refers to eligibility criteria requiring an ASD diagnosis.

^d^ Refers to home- and community-based service waivers that are for individuals with intellectual disability or developmental disability. This may include individuals with ASD, but eligibility does not include ASD specifically.

The annual probability of disenrollment (Wald *P* < .001) (Figure) was similar among individuals in states with an ASD-specific waiver, other waiver, or no waiver through age 21 years. Disenrollment increased at age 19 years among those in states with waivers but was less pronounced in states without waivers. At age 22 years, the probability of disenrollment in states without waivers increased markedly (>13%) and remained elevated through age 26 years. In states with a waiver, probability of disenrollment remained stable (approximately 2%) for the same age range.

**Figure.**
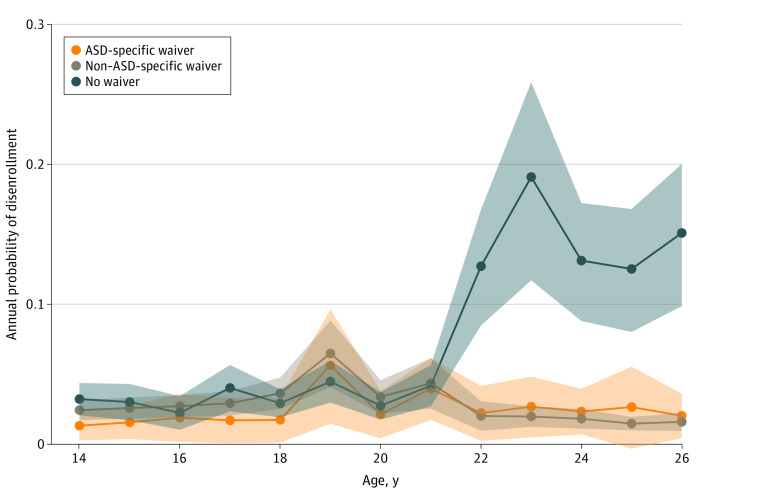
Adjusted Annual Probability of First Disenrollment Among Medicaid Beneficiaries With Autism Spectrum Disorder (ASD) During the Period 2008 to 2016 by Waiver Availability Shaded areas represent 95% CIs.

## Discussion

Residence in a state with Medicaid waivers was associated with a 6-fold or greater decrease in probability of disenrollment among autistic adolescents entering adulthood. These findings are important for state policy, as being insured is associated with fewer unmet health care needs^4,5^ and may reduce inpatient and long-term care services and expenses.^6^ States without waivers available for autistic young adults should consider implementing or extending existing waivers to maintain insurance coverage and subsequently improve health outcomes. Interestingly, ASD-specific waivers were not associated with greater decreases in disenrollment compared with waivers for other diagnoses or functional eligibility criteria, suggesting that ASD-specific waivers may not mitigate disenrollment. Our findings suggest that requirements for remaining continuously enrolled in Medicaid when poverty-eligible may be challenging, as small changes in income may result in disenrollment.

Study limitations include the potential for misclassification due to incomplete or missing claims data. Policy makers should consider bolstering continuity of waiver availability to keep autistic youths insured and connected to necessary health care services or explore other eligibility mechanisms for Medicaid.^2^

## Notes

Supplement 1.
eMethods.

eReferences

Supplement 2.
Data Sharing Statement

## References

[1] Shea L, Tao S, Marcus SC, Mandell D, Epstein AJ. Medicaid disruption among transition-age youth on the autism spectrum. Med Care Res Rev. 2022;79(4):525-534. doi:10.1177/10775587211051185 34632834 PMC10775849

[2] Shea LL, Koffer Miller KH, Verstreate K, Tao S, Mandell D. States’ use of Medicaid to meet the needs of autistic individuals. Health Serv Res. 2021;56(6):1207-1214. doi:10.1111/1475-6773.13671 34251042 PMC8586478

[3] Grosse SD, Nichols P, Nyarko K, Maenner M, Danielson ML, Shea L. Heterogeneity in autism spectrum disorder case-finding algorithms in United States health administrative database analyses. J Autism Dev Disord. 2021;52(9):4150-4163. doi:10.1007/s10803-021-05269-1 34581918 PMC9077262

[4] Karpur A, Lello A, Frazier T, Dixon PJ, Shih AJ. Health disparities among children with autism spectrum disorders: analysis of the National Survey of Children’s Health 2016. J Autism Dev Disord. 2019;49(4):1652-1664. doi:10.1007/s10803-018-3862-9 30552540

[5] Barry CL, Epstein AJ, Marcus SC, . Effects of state insurance mandates on health care use and spending for autism spectrum disorder. Health Aff (Millwood). 2017;36(10):1754-1761. doi:10.1377/hlthaff.2017.0515 28971920 PMC7011707

[6] Cidav Z, Marcus SC, Mandell DS. Home- and community-based waivers for children with autism: effects on service use and costs. Intellect Dev Disabil. 2014;52(4):239-248. doi:10.1352/1934-9556-52.4.239 25061768 PMC4769871

