# Errors in Results

**DOI:** 10.1001/jamanetworkopen.2023.15445

**Published:** 2023-05-10

In the Research Letter titled “Association Between Medicaid Waivers and Medicaid Disenrollment Among Autistic Adolescents During the Transition to Adulthood,”^1^ published March 13, 2023, certain percentages that appeared in the first paragraph of the Results did not match the corresponding percentages in the Table. This article has been corrected.
