# Supplementary material for: Association Between Medicaid Waivers and Medicaid Disenrollment Among Autistic Adolescents During the Transition to Adulthood
Source: JAMA Netw Open. 2023 Mar 13;6(3):e232768. doi: 10.1001/jamanetworkopen.2023.2768 (PMC10011936; doi:10.1001/jamanetworkopen.2023.2768)
Supplement: Supplement 1. — eMethods. eReferences [file jamanetwopen-e232768-s001.pdf]

Supplemental Online Content

Carey ME, Tao S, Koffer Miller KH, et al. Association between Medicaid waivers and Medicaid disenrollment among autistic adolescents during the transition to adulthood. *JAMA Netw Open*. 2023;6(3):e232768. doi:10.1001/jamanetworkopen.2023.2768

eMethods.
eReferences

This supplemental material has been provided by the authors to give readers additional information about their work.

© 2023 Carey ME et al. *JAMA Network Open.*

eMethods

Study design and data sources

We conducted a retrospective cohort study of Medicaid beneficiaries using data from the national Centers for Medicare and Medicaid Services (CMS). Data were extracted from the 2008–2015 Medicaid Analytic eXtract (MAX) and 2014-2016 Transformed Medicaid Statistical Information System Analytic Files (TAF) personal summary and service files. Autistic individuals were identified using diagnostic codes consistent with established Medicaid algorithms (299.xx [ICD-9-CM] or F84.x [ICD-10-CM]). We conducted a secondary data analysis that did not require active participation by Medicaid enrollees. As such, informed consent was not obtained from study participants. This study followed STROBE reporting guidelines.

Variables

Outcome: Disenrollment

The outcome was a binary indicator for first-observed disenrollment for a given individual in a given month. We specify first-observed disenrollment because individuals may have experienced disenrollment not captured in this analysis (i.e., before follow-up period or earlier than 14 years old). Disenrollment was measured as a gap in coverage of ≥2 consecutive months. Disenrollment for administrative reasons, referred to as administrative churning, is common in Medicaid and may result in coverage disruptions for one or two months.[1]

Exposure: Waiver availability

The exposure was a categorical measure for whether a waiver was available in the state a given individual resided in for a given month (mutually exclusive categories: ASD-specific waiver, other waiver, no waiver). Waivers to Medicaid programs are provided at the state level. Some states will have multiple waivers simultaneously. Three states (Arizona, Vermont, and Rhode Island) use 1115 research and demonstration projects which are broader in scope.[3,4] Our analysis focused on the 1915(c) waiver mechanism as: 1) Most states provide services to autistic youth this way;[2] and 2) there is more uniformity in eligibility and service coverage compared to the 1115 mechanism. ASD-specific waivers refer to 1915(c) waivers that specifically list diagnostic eligibility criteria for ASD.[3]

Covariates

Individual-level covariates measured during the eligibility period included sex (male, female), race and ethnicity (American Indian or Alaska Native, Asian or Hawaiian or other Pacific Islander, Black, Hispanic or Latino, White, Multiracial, missing). Additional individual-level covariates were measured on a monthly basis and included age (treated as an integer for models; categorized as 14-17, 18-21 and 22-26 in Table 1), Medicaid eligibility group (poverty, disability, other, missing), Medicaid coverage type (fee-for-service/primary care case management [FFS/CCM] only, any comprehensive managed care [CMC]), Medicare dual enrollment (yes/no). As part of the enrollment process, Medicaid asks for self-reported race and ethnicity; however, this information is not used in eligibility determination and optional to report, occasionally resulting in missing observations. No imputation was conducted for missing variables per CMS recommendation.[5] Instead, a missing category was created for these cases.

Statistical Analysis

The risk of disenrollment was assessed using a person-month discrete-time proportional hazards model framework. The model specification included main effects for age, state-level waiver availability and their interaction, and it controlled for sex, race and ethnicity, Medicaid eligibility category, and Medicaid coverage type. Vectors of binary indicators for state of residence and calendar year were included in the model as fixed effects. The state fixed effects control for time-invariant state-level factors. With the state and year fixed effects, identification of the association between waiver availability and disenrollment was based on variation in exposure and outcome within states over time. Clustering of standard errors by state in conjunction with fixed effects for state is common.[6] Individuals were considered to be at risk of disenrollment beginning in the first month after their eligibility period until their first disenrollment event, until they reached age 26, or until the time of censoring in December 2016.

Identification of the association between waiver availability and disenrollment was based on variation in exposure and outcome within states over time. The discrete-time hazard model was estimated via maximum likelihood complementary-log-log regression.[7] Model standard errors were adjusted to be robust to heteroskedasticity of unknown form and clustering of individuals within states.[8] Marginal predicted hazard rates and

© 2023 Carey ME et al. *JAMA Network Open.*

their 95% confidence intervals (CIs) were calculated for each of the waiver groups by age. To ease interpretation, the month-level hazard rates and 95% CIs were converted into annual probabilities of disenrollment using the formula $p = 1 - \exp(rt)$, where p represents the probability, r is the predicted hazard rate and t is set to 12 months.[9] Analyses were conducted using Stata 16.1 (College Station, TX) and R 4.2.0. Statistical tests used a 2-sided α of .05.

eReferences

1. Shea LL, Tao S, Marcus SC, Mandell D, Epstein AJ. Medicaid Disruption Among Transition-Age Youth on the Autism Spectrum. *Medical Care Research and Review.* 2022;79(4):525-534.
2. Schneider A, Elias R, Garfield R. Medicaid eligibility. *The Medicaid Resource Book.* 2003:17-18.
3. Shea LL, Koffer Miller KH, Verstreate K, Tao S, Mandell D. States' use of Medicaid to meet the needs of autistic individuals. *Health Services Research.* 2021;56(6):1207-1214.
4. Medicaid and CHIP Payment and Access Commission (MACPAC). Section 1115 research and demonstration waivers. https://www.macpac.gov/subtopic/section-1115-research-and-demonstration-waivers/. Accessed 12 December, 2022.
5. Saunders H, Chidambaram P. *Medicaid Administrative Data: Challenges with Race, Ethnicity, and Other Demographic Variables.* https://www.kff.org/medicaid/issue-brief/medicaid-administrative-data-challenges-with-race-ethnicity-and-other-demographic-variables/: KFF; Apr 28 2022.
6. Cameron AC, Miller DL. A practitioner’s guide to cluster-robust inference. *Journal of human resources.* 2015;50(2):317-372.
7. Jenkins SP. Easy estimation methods for discrete-time duration models. *Oxford bulletin of economics and statistics.* 1995;57(1):129-138.
8. Williams RL. A note on robust variance estimation for cluster-correlated data. *Biometrics.* 2000;56(2):645-646.
9. Briggs A, Sculpher M, Claxton K. *Decision modelling for health economic evaluation.* Oup Oxford; 2006.

© 2023 Carey ME et al. *JAMA Network Open.*
