# Supplementary material for: Association Between Medicaid Waivers and Medicaid Disenrollment Among Autistic Adolescents During the Transition to Adulthood
Source: JAMA Netw Open. 2023 Mar 13;6(3):e232768. doi: 10.1001/jamanetworkopen.2023.2768 (PMC10011936; doi:10.1001/jamanetworkopen.2023.2768)
Supplement: Supplement 2. — Data Sharing Statement [file jamanetwopen-e232768-s002.pdf]

Data Sharing Statement

Carey. Association Between Medicaid Waivers and Medicaid Disenrollment Among Autistic Adolescents During the Transition to Adulthood. *JAMA Netw Open*. Published March 13, 2023. doi:10.1001/jamanetworkopen.2023.2768

Data
Data available: No

Additional Information
Explanation for why data not available: The data used in this analysis comes from the Centers for Medicare and Medicaid Services and we do not own it.
